# Clinical application of a modified percutaneous vertebroplasty instrument in vertebral body biopsy in adults

**DOI:** 10.1186/s12891-022-05117-y

**Published:** 2022-02-19

**Authors:** Fubiao Zhou, Ningkui Niu, Qiang Liang, Yueliang Chang, Jiandang Shi, Zili Wang

**Affiliations:** 1 Department of Hand Surgery, The No. 971 Hospital of People’s Liberation Army Navy, Qingdao, China; 2 Department of Spinal Surgery, General Hospital of Ningxia Medical University, 804, Shengli Street, Yinchuan, 750004 China; 3 Department of Spinal Surgery, Yantai yuhuangding hospital, Yantai, China

**Keywords:** CT-guided, Modified PVP instrument, Biopsy, Clinical application

## Abstract

**Background:**

Percutaneous vertebroplasty (PVP) has been widely used to treat vertebral pathological fractures in recent decades, and the modified PVP instrument is very suitable for percutaneous biopsy of diseases promoting vertebral bone destruction. The purpose of this study was to evaluate the relevance of the clinical application of the modified PVP instrument in computed tomography-guided (CT-guided) biopsies of the vertebral body.

**Methods:**

Retrospective analysis of clinical data obtained by percutaneous biopsy using a modified PVP outer shell of a bone filler device (OSBF) from 161 patients presenting vertebral body destruction was conducted. The rate of correctly performed biopsy diagnosis was evaluated from three aspects: imaging performance, histological type, and vertebral segment.

**Results:**

The results of 149 biopsy cases were consistent with the final clinical diagnosis. From those cases, 92 were diagnosed as vertebral body metastasis, 45 cases presented primary spinal tumors and tumor-like changes, 7 cases presented vertebral body infections, and 5 cases displayed normal bones or fractures. From the remaining 12 patients, whose biopsy results were inconsistent with the final clinical diagnosis, 4 presented vertebral metastases, 4 displayed primary vertebral tumors, and 4 presented vertebral infections. The diagnostic rate of the modified PVP OSBF biopsy was 92.5%. The rate of correct biopsy diagnosis for vertebral metastases was 95.8%. The rate of correct diagnosis of primary vertebral tumors and tumor-like biopsy was 91.8%, and the rate of correct diagnosis for vertebral infectious diseases was 63.6%.

**Conclusion:**

The modified PVP OSBF allows obtaining more lesion tissue, in multiple directions and multiple angles, during the biopsy of vertebral bones presenting destructive lesions. The technique displays appropriate safety and high diagnostic accuracy and presents a desirable reference value for the preoperative diagnosis of diseases that yield vertebral bone destruction, especially for vertebral tumor lesions.

## Background

Pathological diagnosis plays an important role in the identification of diseases promoting vertebral bone destruction. Compared with incisional biopsy, CT-guided percutaneous biopsy has become a common method for preoperative diagnosis of destructive lesions in the vertebral bone because of its safety, simple operation, and a smaller number of complications associated [1, 2]. Currently, fine needles and percutaneous trocars are commonly used in the clinical practice. In the case of fine needle aspiration biopsy, the amount of tissue acquired is too small, especially for hardened bone lesions, which is difficult to obtain, rendering diagnosis difficult [3, 4]. In the case of percutaneous trocar biopsy, although the amount of specimen obtained is sufficient, if the specimen obtained for the first time is not ideal, it is necessary to reposition the instrument and reestablish the puncture channel to perform the procedure again, consuming both time and energy [5, 6].

In the clinical practice, we found that the modified PVP OSBF is suitable for transpedicular biopsies. We employed it by using the grinding technology to transform the smooth edge of the OSBF into a jagged edge, allowing it to rotate in the vertebral body under the CT guidance, thus, cutting the lesion and adjusting the channel direction and puncture depth according to the lesion position. Thereby, it allows to repeatedly obtain the lesion tissue until a satisfactory sample is attained. This article retrospectively analyzed the clinical data of 161 patients presenting vertebral bone destruction who underwent CT-guided percutaneous biopsy using the modified PVP OSBF from January 2010 to December 2019, in the Department of Spinal Orthopaedics of the Ningxia Medical University General Hospital. We aimed at exploring the clinical application validity of the puncture device in diseases that promote vertebral bone destruction.

## Methods

### General Information

Clinical data of 161 patients, who were treated for vertebral bone destruction in the Department of Spinal Orthopaedics, General Hospital of Ningxia Medical University from January 2010 to December 2019, was collected. There were 87 males and 74 females, aged 18-78 years, with the mean age of 47.71±14.23 years, and all patients signed an informed consent form. Spinal X-ray and CT were performed before the operation and different degrees of vertebral bone destruction were observed.

### Puncture equipment

**Fig. 1:**
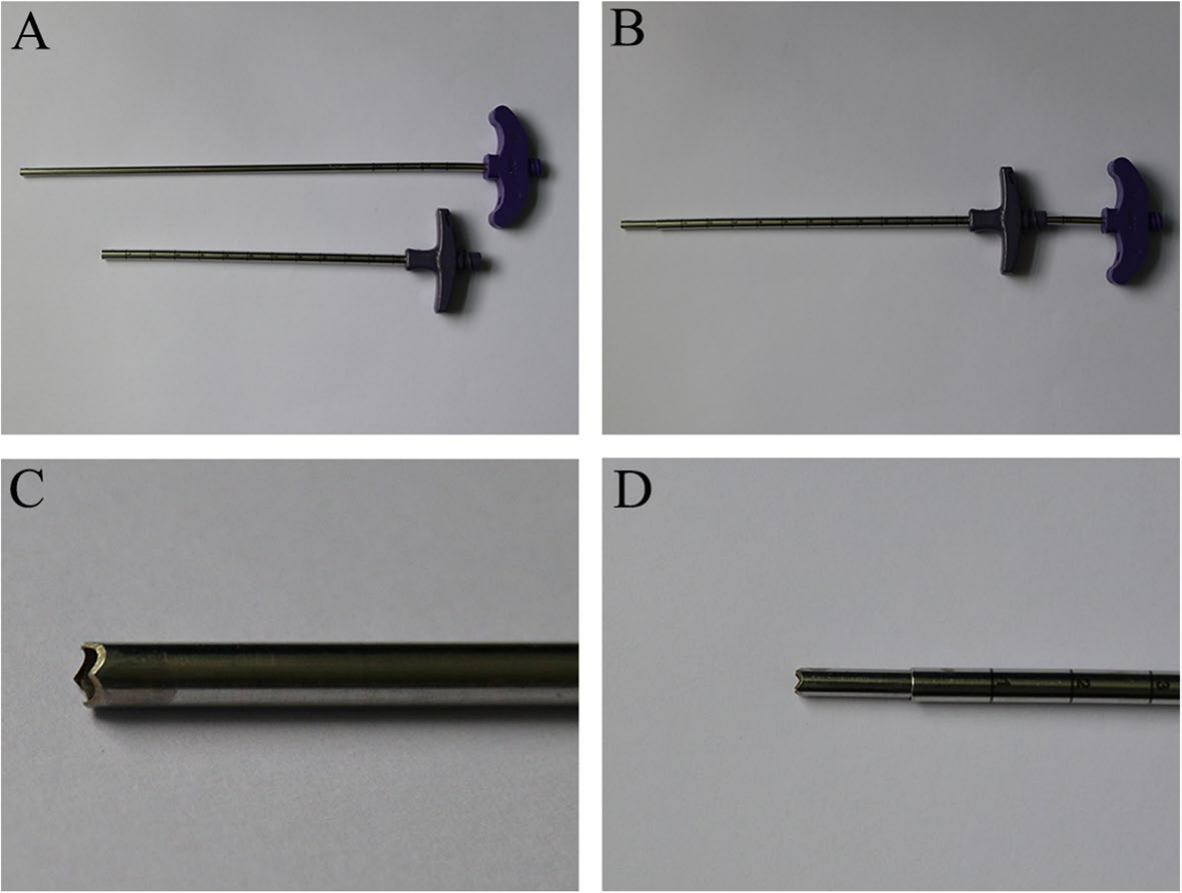
Modified puncture device. **A** Modified OSBF (pipe diameter: 3.4/3.6 mm,length: 195 mm) and casing channel (pipe diameter: 4.0/4.2 mm,length: 125 mm); **B** Modified OSBF combined with casing channel; **C** Jagged edges of modified OSBF; **D **Distal-end appearance after the modified OSBF and casing channel combination

The equipment included a Siemens 64-row spiral CT machine, a disposable chest puncture package, normal saline solution, 2% lidocaine injection, heparin sodium injection, the modified OSBF, and the related working channels (Fig. 1).

### Preparation before puncture

The patients presented a good general condition before the surgery and the prone position could be maintained for more than 30 minutes. The routine blood exams and coagulation function were normal and the skin of the puncture site was intact. The imaging examination of the lesion site was completed prior to the puncture, and the puncture site should be located on the subsequent surgical incision as apart as possible. The patients signed the informed consent form for the biopsy.

### Puncture procedure

**Fig. 2:**
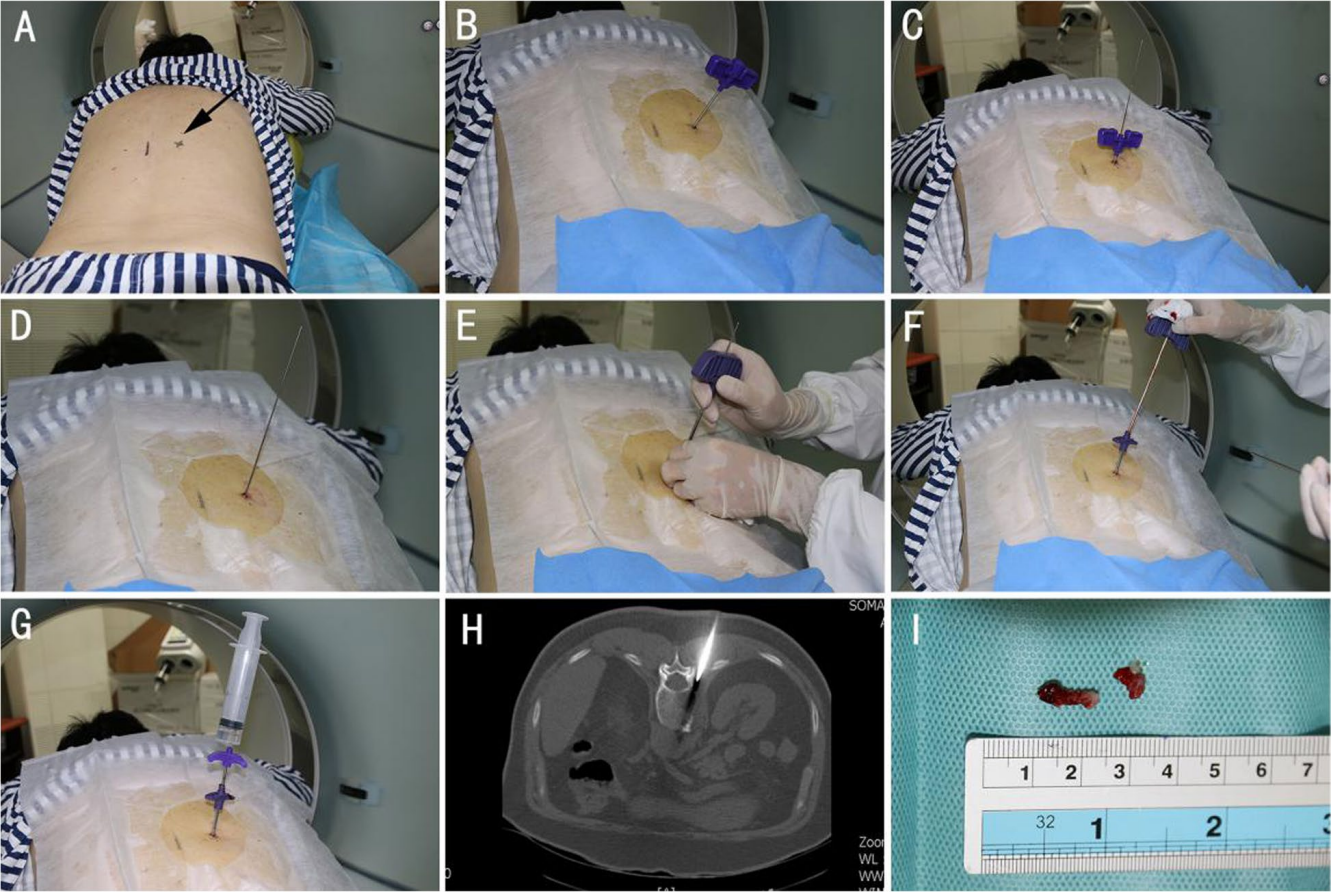
62-year-old male, lumbar MRI suggests the first lumbar vertebral metastases. The above figure shows the process of lumbar puncture biopsy using modified OSBF. **A** Puncture point positioning under CT guidance before operation; **B**-**D** Insert the guide needle; **E** Insert the casing channel; **F** Insert modified OSBF; **G** Under Vacuum Suction, the modified OSBF rotates forward and cuts the lesion; **H** Intraoperative CT guided positioning; **I** Specimen obtained during puncture

**Fig. 3:**
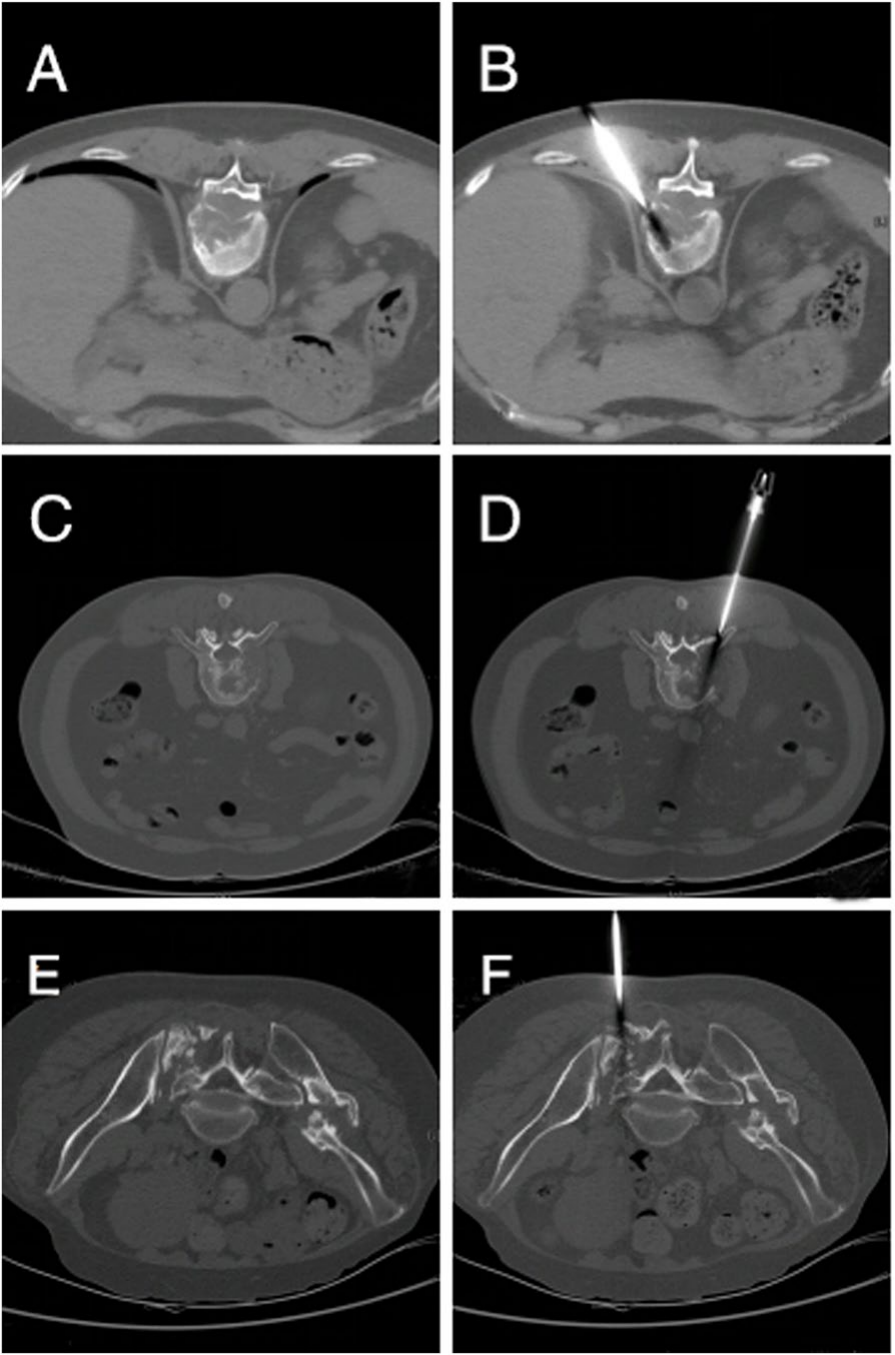
Vertebroscopic biopsy under CT guidance. **A** and **B**. Puncture biopsy of a solitary plasmacytoma in the eleventh thoracic vertebra; (**C** and **D**) Puncture biopsy of an undifferentiated pleomorphic sarcoma of the body of the third lumbar vertebra; (**E** and **F**) Puncture biopsy of a sacral metastasis of a right kidney clear-cell carcinoma

The transpedicular approach was routinely selected and the measurement marks of the puncture site were performed under CT guidance before surgery to determine the point and angle for inserting the needle. We performed iodine disinfection of the puncture point and 2% lidocaine local anesthesia with a long needle for local anesthesia of the pedicle periosteum. A longitudinal incision in the skin of circa 0.7cm was performed, the needle with its core enters at the predetermined angle and direction, after entering the pedicle, the needle is withdrawn and placed into the casing channel according to the relevant steps of the vertebroplasty. The modified OSBF is inserted into the casing channel and fixed, the tail end is connected to a 60 mL syringe under vacuum suction to slowly enter the lesion, and the process of rotating the serrated cutting edge may obtain a columnar structure conforming to the inner diameter of the OSBF. The modified OSBF was removed, but the casing channel was retained. The removed sample was placed in a heparin saline solution, filtered through a gauze, and the lesion tissue was placed in 4% formalin for pathological examination. Those suspected of presenting a vertebral body infection were also sent for pathogen examination. If the lesion tissue is not acquired or insufficiently obtained, the angle and depth of the casing channel were adjusted and the lesion tissue was repeatedly drilled until a satisfactory sample was obtained (Figs. 2 and 3). When the sample was sufficient for pathological examination, the needle was withdrawn and the puncture site was pressed to stop bleeding and covered with a sterile dressing.

### Evaluation method

The pathological diagnosis of needle biopsy was made by three pathologists in the hospital. The patient's final diagnosis was based on the patient's clinical presentation, imaging exams, laboratory tests, postoperative pathology, response to treatment, and follow-up. The diagnosis was determined as correct when the pathological or pathogenic diagnosis of the puncture sample was identical to the final clinical diagnosis of the patient.

## Results

All 161 patients were successfully punctured without severe puncture complications and sufficient samples were obtained. There were 3 patients with lower extremities radioactive pain during the PVP OSBF puncture biopsy, and the symptoms were relieved when transforming the puncture needle position and angle;3 patients had obvious low back pain after the biopsy, but their symptoms were relieved after 1 weeks of waist brace;In addition, 2 patients had hematomas around the puncture point, and the symptoms disappeared after the gauze was compressed. The remaining patients had no obvious complications. We performed biopsies on 64 cases of thoracic vertebrae lesions, 73 cases of lumbar vertebrae lesions, and 24 cases of sacral lesions (Table 1). The puncture duration (including preoperative CT scan) was 21-39 min (27.4±3.7 min), and the mean blood loss was of 5 mL. The skin incision healed well after surgery, without bleeding and wound infection. None of the patients presented damage to the spinal cord, nerves, large blood vessels or important organs during and after needle biopsy.

**Table 1:** The location and number of vertebral bodies punctured

Thoracic vertebrae (64)	Lumbar vertebrae (73)	Sacral vertebrae (24)
T1(2)	L1(13)	S1(12)
T3(3)	L2(12)	S2(6)
T5(5)	L3(15)	S3(6)
T6(3)	L4(18)	
T7(7)	L5(15)	
T8(7)		
T9(9)		
T10(5)		
T11(11)		
T12(12)		

*T* Thoracic vertebrae, *L* Lumbar vertebrae, *S* Sacral vertebrae

The diagnosis of needle biopsy in 149 patients was consistent with the final clinical diagnosis, which included 92 patients with vertebral metastases, 45 patients with primary spinal tumors and tumor-like changes, 7 patients with vertebral infection, and 5 patients with normal bone or fracture. Among them, 66 patients underwent further surgeries for the following conditions: 1) 25 cases for vertebral metastases; 2) 38 cases for primary tumors and tumor-like changes, i.e. 8 cases of giant cell tumor of bone, 5 cases of osteosarcoma, 2 cases of chordoma, 6 cases of osteochondroma, 3 cases of chondrosarcoma, 3 cases of myeloma, 2 cases of solitary plasmacytoma, 1 case of osteoblastoma, 4 cases of aneurysmal bone cyst, and 4 cases of osteofibrous dysplasia; and 3) 3 cases for vertebral body infections, i.e. 2 cases of spinal tuberculosis and 1 case of suppurative spondylitis. From those patients, 11 patients with vertebral metastases underwent vertebroplasty, and no surgical sampling was performed. The pathological diagnosis of the remaining 55 patients was consistent with the needle biopsy diagnosis. A total of 83 patients were not treated with surgery, including 67 cases of vertebral metastases, 1 case of Ewing's sarcoma, 4 cases of myeloma, 2 cases of lymphoma, 4 cases of brucella spondylitis,and 5 cases of normal bone or fracture. For the 5 patients with negative puncture results, the biopsy pathological analysis of 2 of them displayed broken bone and fibrous tissue, which, according to the clinical follow-up, was diagnosed as an osteoporotic ftacture. For 2 patients, the biopsy pathology showed blood clots, bone marrow tissue, bone and hyaline cartilage; this was correlated in the clinical follow-up to stress fractures. One patient presented no characteristic changes in the pathological results, according to clinical follow-up; the diagnosis was an old compression fracture. For the 83 patients treated non-surgically, the final diagnosis was made by three clinically experienced chief physicians based on clinical data, conservative treatment response, and follow-up. The final diagnosis was consistent with the puncture diagnosis.

The biopsy diagnosis of 12 patients was inconsistent with the final clinical diagnosis. Four patients were diagnosed with vertebral metastases based on clinical follow-up. One of the patients underwent breast cancer surgery; her bone scintigraphy imaging showed metastatic changes in multiple ribs and in the seventh thoracic vertebral body. One patient was diagnosed with lung squamous cell carcinoma by fiberoptic bronchoscopy and the lumbar CT showed possible metastasis in the third lumbar vertebra. In two patients presenting postoperative esophageal cancer, the bone scintigraphy showed multiple bone metastases in the body. The above four patients did not operate surgery due to no surgical indications. According to the primary tumor properties, clinical symptoms, bone scanning results, patients finally diagnose metastatic cancer. However, no tumor cells were seen in the needle biopsy of the four patients mentioned above. The biopsy results from the first puncture in four patients were negative; two of those patients underwent surgery and their postoperative pathological diagnosis was osteosarcoma and giant cell tumor of bone, and the two remaining patients underwent a second biopsy two weeks later, which yielded the final diagnosis of chondrosarcoma and osteoblastoma. For further four patients, the biopsy only showed inflammatory cell infiltration, but the pathogen test was negative. 3 patients’ T-Spot examinations were positive, and they were processed by surgery, 4-6 months of anti-tuberculosis drug treatment; 1 patient’s brucella agglutination test was positive, who took medicine for treatment. 6 months follow-up, 4 patients’ low back pain disappeared. Therefore, based on the patient's clinical data and response to empirical treatment, the final diagnosis of three patients was spinal tuberculosis and that of one patient was brucella spondylitis.

The imaging data of 161 patients were analyzed (Table 2). There were 74 cases of osteolytic bone destruction, and the accuracy of the biopsy diagnosis was of 93.2% (69/74). There were 38 cases of sclerosing bone destruction, and the accuracy of the biopsy diagnosis was of 92.1% (35/38). There were 34 cases of mixed bone destruction, for which the biopsy diagnosis was consistent with the final diagnosis. There were 15 cases of atypical bone destruction, for which the accuracy of biopsy diagnosis in this part of patients was of 73.3% (11/15).

**Table 2:** Diagnostic accuracy of the biopsy for different classes of bone destruction

Bone destruction properties	Number of cases	Correct diagnosis	Accuracy rate
Osteolytic	74	69	93.2%
Sclerosing	38	35	92.1%
Mixed	34	34	100%
Atypical	15	11	73.3%
total	161	149	92.5%

The different types of diseases observed in the 161 patients are presented in Table 3. There were 96 cases of vertebral metastases, from which, 92 cases were correctly diagnosed by the biopsy, yielding a diagnostic accuracy of 95.8%. Primary tumors and tumor-like changes in the spine accounted for 49 cases, from which 45 cases were correctly diagnosed by the biopsy, and the diagnostic accuracy was 91.8%. There were 11 cases of vertebral body infection, from which 7 cases were correctly diagnosed by pathogen analysis, yielding a diagnostic accuracy rate of 63.6%. The other five cases were confirmed as fractures by clinical follow-up.

**Table 3:** Diagnostic accuracy of the biopsy for different disease types

Histological type	Disease (undiagnosed / total number of cases)	Correct diagnosis	Diagnostic error	Correct rate
Vertebral metastases		92	4	95.8%
Primary tumor and tumor-like change		45	4	91.8%
	Ewing's sarcoma (1)			
	Giant cell tumor of bone (1/9)			
	Osteosarcoma (1/6)			
	Chordoma (2)			
	Osteoblastoma (1/2)			
	Osteochondroma (6)			
	Chondrosarcoma (1/4)			
	Aneurysmal bone cyst (4)			
	Myeloma (7)			
	Isolated plasmacytoma (2)			
	Lymphoma (2)			
	osteofibrous dysplasia (4)			
Vertebral infection		7	4	63.6%
	spinal tuberculosis(3/5)			
	brucella spondylitis(1/5)			
	suppurative spondylitis(1)			
Normal bone or fracture		5		100%
total		149	12	92.5%

Table 4 summarizes the vertebral segment punctured in the 161 patients. A total of 64 patients underwent thoracic puncture, for which the accuracy of the biopsy was of 93.8% (60/64). Seventy patients underwent lumbar puncture and the accuracy of the biopsy was of 94.5% (69/73). Further 24 patients underwent sacral puncture, presenting an accuracy of the biopsy of 83.3% (20/24).

**Table 4:** Accuracy of the needle biopsy for different vertebral segments

Vertebral body	Number of puncture cases	Correct diagnosis	Accuracy rate
Thoracic vertebra	64	60	93.8%
Lumbar vertebra	73	69	94.5%
Sacral vertebra	24	20	83.3%
total	161	149	92.5%

## Discussion

Pathological diagnosis plays an important role in the assessment of destructive bone lesions. Before the treatment, a certain amount of tissue is obtained through needle biopsy for pathological diagnosis, which is of major significance for the selection of treatment plan and efficacy evaluation [1, 2, 7, 8]. Pathological specimens are generally obtained by incisional biopsy and needle biopsy. Although the accuracy of incisional biopsy is high and it allows obtaining a sufficient amount of lesion tissue [9], it presents several hindrances, e.g. high surgical cost, large trauma, and contamination of the surrounding normal tissue by the lesions [7]. Compared with incisional biopsy, percutaneous biopsy has become the main method for preoperative biopsy of vertebral bone destruction diseases because of its simple operation, a smaller number of complications, and high accuracy [10]. However, there are still many problems regarding the previous puncture methods, e.g. insufficient collection of lesion tissue, a greater number of complications, high cost, and low diagnostic accuracy [11–17].

Based on previous clinical experience, our team found that the modified OSBF is suitable for the biopsy of vertebral destruction lesions, and may significantly improve the shortcomings of traditional biopsy methods. Currently, PVP is widely used to treat vertebral osteoporotic compression fractures [18–20], and its matching threaded rod may be used to obtain the lesion tissue of the vertebral body [21, 22]. However, only a small amount of lesion tissue might be obtained through the gap of the thread, which cannot be used for biopsy. The diameter of the OSBF in the PVP is relatively large. Therefore, if it is employable for a needle biopsy, a more significant sample of the lesion may be obtained. Thus, we used the grinding technique to polish the smooth edge of OSBF into a jagged edge and it was successfully used for a needle biopsy in diseases associated with vertebral destruction.

In this study, the accuracy of needle biopsy in patients with primary tumors and tumor-like changes in the spine was of 91.8%, which was smaller than the accuracy of needle biopsy in patients with vertebral metastases (95.8%), these results are similar to what is found in the current literature [23–27]. For the primary tumor and tumor-like changes of the spine, the lesion site often lacks typical pathological features so that the diagnosis requires an increasing amount of typical lesion tissue [28, 29]. For vertebral metastases, pathologists have already known the possible malignant tumors and the source of the lesions [27], which is also the objective reason why many researchers believe that the accuracy of the tumor and tumor-like changes of the spine is lower than that of the vertebral metastases. Kamei et al [26] performed needle biopsies on 128 patients. The accuracy rate of primary tumor biopsy was 78.6%, and the accuracy of biopsy of metastatic tumors was 97.0%. Yang et al [27] performed needle biopsies on 247 patients. Needle biopsies yielded an accuracy of 84% for primary tumors and 97% for metastatic cancer. We used a modified OSBF to perform biopsies on 49 patients with primary tumor and tumor-like changes in the spine. The biopsy diagnosis of 45 patients was consistent with the final clinical diagnosis, which resulted in an accuracy rate of 91.8%, higher than that in previous studies.

The lesions found in vertebral infectious diseases are basically non-specific considering pathological manifestations, only showing the infiltration of a large number of inflammatory cells and no tumor cells. Granuloma and caseous necrosis are sometimes found in the lesions of some patients with specific vertebral infections. Therefore, the pathological manifestations of puncture specimens in patients with spinal infections may only be used as a reference for final diagnosis. We routinely perform pathogen examinations on patients suspected of having an infection before the operation. 11 patients in this study presented spinal infections, from which, 7 were positive for puncture pathogen culture, with a diagnostic accuracy of 63.6%. Eugenio et al [30] performed needle biopsies on 31 patients with spinal infection and the diagnostic accuracy rate is 58%, their results are similar to those found in our research. The timing of specimen collection, storage after collection, time of inspection, different test methods, and abuse of broad-spectrum antibiotics all lead to low culture positive rates [13]. Therefore, the diagnostic accuracy of needle biopsy in those cases is lower than that of spinal tumors.

Previous studies with CT-guided vertebral biopsy have found that the diagnostic accuracy for sclerosing lesion biopsy is significantly lower than for osteolytic lesions [27, 31, 32]. An analysis of the reason is that for sclerosing lesions, there is hyperplasia of the vertebral cortical bone, therefore, traditional puncture instruments usually may not be able to penetrate the cortical bone smoothly. When compared with osteolytic lesions, sufficient and typical lesion tissue might not be obtained, so that the diagnostic accuracy of the needle biopsy is low. We modified the OSBF used in PVP to sharpen its smooth edges into sharp jagged edges while preserving the rigidity and strength of the serrations, ensuring that the modified OSBF may easily rotate and cut the hyperplastic cortical bone, and can reach the center of the lesion. Therefore, in our study, the diagnostic accuracy in osteolytic lesions was of 93.2%, while the diagnostic accuracy of sclerosing lesions was of 92.1%. There was no statistically significant difference between the two but both were considered to be of a high level.

It is reported that the accuracy of CT-guided biopsy diagnosis is lower in the thoracic spine [33]. However, in our study, the accuracy rates of the thoracic and lumbar biopsy were 93.8% and 94.5%, respectively, both of which were at a high level. This finding confirmed that the modified OSBF has a high value for application in the biopsy of diseases promoting vertebral destruction. The accuracy of the biopsy diagnosis in the sacral vertebrae is 83.3%, which was considered to be low in our study. The reason may be that metastases are more common in the thoracic vertebrae and lumbar vertebrae, and the primary tumors are more common in the sacral vertebrae. As previously mentioned, the accuracy of biopsies of spinal primary tumors and tumor-like biopsies is lower than that of spinal metastases.

Guo et al [31] performed needle biopsies on 171 patients with bone tumors under the guidance of imaging techniques. The final biopsy accuracy rate was of 80.33%. Wu et al [24] performed needle biopsies on 151 patients presenting diseases that promote bone destruction guided by guidance imaging techniques and the final diagnostic accuracy was of 77%. Finally, Yang et al [27] performed needle biopsies on 247 patients with vertebral lesions under the guidance of imaging tools and 197 cases (80%) were correctly diagnosed. In the present study, we performed needle biopsies on 152 patients with vertebral bone destruction. A total of 149 patients were correctly diagnosed. The diagnostic accuracy was of 92.5%, which was considered to be of a high level. Because when we use the modified OSBF puncture, we were able to adjust the angle and depth of the cannula channel according to the intraoperative CT image to drill the lesion multiple times. Moreover, the modified OSBF has a larger diameter, and the strip lesion tissue may be obtained after each drilling. Therefore, we could obtain a sufficient amount of typical lesion sample, which is the reason for the high accuracy in our biopsy diagnosis.

For some patients in this study, the best timing for surgery was lost at the time of the visit, resulting in the necessity of radiotherapy and chemotherapy. Some patients with vertebral metastases abandoned the treatment. Although patients who had not undergone surgery had no postoperative pathological diagnosis, a final diagnosis could be made based on the patient's clinical data, response to treatment, and follow-up. Due to the complexity of the tissue structure around the vertebral body, the surgeon of the puncture biopsy should be skilled in the spine anatomy. The puncture should be carried out under the accuracy of the CT, avoid damage to the blood vessels and nerves. The patient's blood regulations and coagulation routine examination should be completed before the puncture biopsy, avoiding the constantly bleeding or hematoma. The surgeon should avoid repeating multiple puncture, reducing the surrounding tissue damage, reducing the low back pain after puncture activity. The puncture device may also be applied to the biopsy of bone tumors in the limbs. The working principle is similar, and the operation method is simpler than those of the standard technique. This study also presents some limitations, e.g. the limited number of cases and no control group, those limitations will be overcome in future researches.

## Conclusion

The modified PVP OSBF presents not only the advantages of traditional percutaneous biopsy needles but also allows obtaining more lesion tissue in multiple directions, furthermore, it displays high safety and high diagnostic accuracy. It has considerable reference value for the preoperative diagnosis of vertebral bone destructive diseases, especially for tumor diseases.

## Data Availability

Readers can access the data and material supporting the conclusions of the study by contacting Fubiao Zhou at zhoufb123@163.com.

## Abbreviations

PVP: Percutaneous vertebroplasty
OSBF: Outer shell of bone filler device
CT: Computed tomography
